# An overview of “Chronic viral infection and cancer, openings for vaccines” virtual symposium of the TechVac Network - December 16-17, 2021

**DOI:** 10.1186/s13027-022-00436-0

**Published:** 2022-07-08

**Authors:** Maria G. Isaguliants, Ivan Trotsenko, Franco M. Buonaguro

**Affiliations:** 1 Riga Stradins University, Riga, Latvia; 2 Department of Microbiology, Tumor and Cell Biology, Karolinska Institutet, Stockholm, Sweden; 3 Peoples Friendship University of Russia (RUDN University), Moscow, Russia; 4 Molecular Biology and Viral Oncology, Department of Experimental Oncology, Istituto Nazionale Tumori Fond Pascale, Naples, Italy

## Abstract

This is a report on the research activities currently ongoing in virology, oncology and virus-associated cancers and possibilities of their treatment and prevention by vaccines and immunotherapies as outlined at the symposium “**Chronic viral infection and cancer, openings for vaccines**” virtually held on December 16–17, 2021. Experts from the various disciplines involved in the study of the complex relationships between solid tumors and viruses met to discuss recent developments in the field and to report their personal contributions to the specified topics. Secondary end point was to sustain the TECHVAC Network established in 2016 as a multidisciplinary work group specifically devoted to development of vaccines and immunotherapies against chronic viral infections and associated cancers, with the aim to identify areas of common interest, promote research cooperation, establish collaborative cross-border programs and projects, and to coordinate clinical and research activities.

## TechVac Meeting, general aspects

The virtual online TechVac Meeting gathered nearly 100 participants from 12 countries: France, Italy, Sweden, Latvia, Germany, Ireland, Spain, Norway, USA, South Africa, South Korea and Russia. The symposium focused on four major topics: Molecular Pathogenesis of Chronic Viral Infections; Chronic Viral Infections and Cancer; Immune Response in Chronic Viral Infections and Cancer; Approaches to Viral Infection and Cancer Cure. All sessions were articulated in 1-2 Plenary Lectures (of 30 min) followed by oral presentations of selected abstracts (of 10 to 20 min). In total, program included 50 presentations including 13 plenary lectures, 28 orals and 9 posters. Symposium was supported by the International Society for Vaccines (ISV), ISV as the partner significantly contributed to the program content, specifically with respect to plenary lectures.

The meeting was opened by Prof Maria Lina Tornesello, Istituto Tumori – IRCCS “Fondazione Pascale”, Napoli, Italy) with a presentation on the *Reversible and Irreversible Activation of Telomerases in HPV-Related Cancers*.

## Session 1: “Molecular pathogenesis of chronic viral infections”

The first session of the symposium was devoted to the molecular pathogenesis of chronic viral infections. It contained two sections, one on chronic viral infections and oxidative stress, and the other on the metabolism of chronic viral infections.

The section on **Chronic viral infections and oxidative stress** was opened by the Plenary Lecture of Alexander Ivanov (Engelhardt Institute of Molecular Biology, Russian Academy of Sciences, Moscow, Russia) on the section topic. The lecture demonstrated that various acute and chronic viral infections are accompanied by enhanced production of reactive oxygen species (ROS), including respiratory viruses, hepatitis B and C viruses (HBV, HCV), human immunodeficiency virus (HIV), human papilloma virus (HPV) and herpesviruses. Levels of oxidative stress markers correlate with virus-associated pathologies: inflammation, fibrosis, and tumor incidence. Moreover, ROS play important roles in the activation of various signaling pathways that promote production of pro-inflammatory cytokines, tumor invasiveness and angiogenesis, adaptation to hypoxic conditions. Changes in redox status and ROS impact replication of many viruses especially in their latency. In recent years intensive efforts were made to unveil sources of ROS that are activated by viruses and to reveal cellular and viral proteins that undergo redox-dependent post-translational modifications. Finally, multi-facetted data have accumulated on interference of viruses with cellular antioxidant defense pathways and the effects of antioxidants that can affect virus replication and suppress development of virus-associated pathologies. With this, increased ROS production during various viral infections stands up as one of the major factors for the development of virus-associated inflammation, fibrosis and cancer. Understanding of the mechanisms by which viruses interfere with redox state of a host cell is critical for the development of approaches for treatment of viral diseases.

The lecture on viral infections and oxidative stress was followed by short presentation of Artemy Fedulov from the same institute on *Hepatitis Delta Virus Antigens Trigger Ros Production and Activate Antioxidant Response Factors.* This study demonstrated that overexpression of HDV antigens or replication of viral genome in Huh 7.5 hepatoma cells altered redox status and enhanced production of superoxide anions both in cytoplasm and mitochondria of the affected cells. The expression of HDV antigens was accompanied by the increased transcription of NADPH oxidases 1 and 4, cytochrome P450 2E1 and ER oxidoreductin 1a (Ero1a) generating both superoxide and hydrogen peroxide. Moreover, expression of small and large delta antigens and HDV RNA replication led to the activation of the transcription factor Nrf2 that controls expression of various antioxidant enzymes. Finally, both HDV antigens triggered unfolded protein response (UPR), as was revealed by increased expression of various UPR-dependent genes. In conclusion, the results showed that HDV antigens alone or in the context of replication of viral RNA trigger ROS production, activate antioxidant Nrf2/ARE pathway and induce unfolded protein response. The input of these processes into HDV pathogenesis calls for further studies.

Kondrashova Alla (Chumakov Federal Scientific Center for Research and Development of Immune and Biological Products of Russian Academy of Science, Moscow, Russia) gave a short presentation focused on *Change of Phenotypical Characteristics of Human Epithelial Cells Made to Overexpress the Reverse Transcriptase (RT) of HIV-1* evaluating the effect of HIV proteins, specifically RT, on epithelial cells infected with HPV. The group has previously shown that RT-expressing adenocarcinoma cells exhibit signs of oxidative stress, express stress-related proteins and demonstrate increased motility and tumorigenicity [1]. These data have given rise to a hypothesis that HIV proteins, including RT, may increase the tumorigenic potential of tumor cells of epithelial origin in HPV-associated tumors. Indeed, the cervical carcinoma cell line CaSki, exposed to exogenous RT, produced ROS, exhibited an increase in E6 expression, but acquired decreased motility. On the contrary to previous observations, CaSki cells stably expressing HIV-1 RT did not produce ROS and did not express oxidative stress related proteins. The motility and clonogenic activity of RT-expressing CaSki were reduced when the level of RT expression was low, however, both properties were rescued in the highly RT-expressing CaSki subclones. The difference in transient and long-term effects of RT could be due to the capacity of HPV16-infected squamous carcinoma cells (SCC) to suppress the production of ROS triggered by RT, including the downstream events. The mechanism behind the rescue of motility and clonogenic activity of SCC by high levels of HIV-1 RT remains to be elucidated.

The second section on **Metabolic signatures of chronic viral infections** was opened by the Plenary Lecture of Birke Bartosch (Cancer Research Center of Lyon, Lyon, France) on *Metabolic Changes in Viral Infections*. A significant portion of cancers in the world is due to chronic viral infections. Viruses induce oncogenesis by targeting the same pathways known to be responsible for neoplasia in tumor cells, such as control of cell cycle progression, cell migration, proliferation and evasion from cell death and the host’s immune defense. In addition, metabolic reprogramming has been identified over a century ago as a requirement for growth of transformed cells. Renewed interest in this topic has emerged recently with the discovery that basically all metabolic changes in tumor cells are finely orchestrated by oncogenes and tumor suppressors. Indeed, cancer cells activate biosynthetic pathways in order to provide them with sufficient levels of energy and building blocks to proliferate and with metabolites that allow cancer cells to modulate the micro-environment in their favor. Interestingly, viruses introduce into their host cells similar metabolic adaptations, and importantly, it seems that they depend on these changes for their persistence and amplification. The central carbon metabolism, for example, is not only frequently altered in tumor cells but also modulated by human papillomavirus, hepatitis B and C viruses, Epstein–Barr virus and Kaposi’s Sarcoma-associated virus. Moreover, adenoviruses and human cytomegalovirus, which are not directly oncogenic but present oncomodulatory properties, also divert cellular metabolism in a tumor cell-like manner. Overall, metabolic reprogramming appears to be a hallmark of viral infection and provides an interesting therapeutic target, in particular, for oncogenic viruses.

In a short presentation on *Trans Cohorts Metabolic Reprogramming Towards Glutaminolysis in Long-Term Successfully Treated HIV-Infection: Potential Role in Accelerated Aging and Latency Reversal* Mikaeloff Flora (Karolinska Institutet, Stockholm, Sweden) reported an altered amino acid (AA) metabolism, more specifically enhanced glutaminolysis, in people living with HIV (PLWH) as compared to healthy controls (HC) which was revealed by the analysis of untargeted and targeted metabolomics. A significantly lower level of neurosteroids was observed in PLWH which could potentiate the neurological impairment observed in HIV infection. Further, modulation of cellular glutaminolysis promoted increased cell death and latency reversal in pre-monocytic HIV-1 latent cell model U1, which may be essential for the clearance of the inducible reservoir in HIV-integrated cells. In conclusion, the study by Mikaeloff et al indicated altered AA metabolism with a switch in glutaminolysis as the alternative pathway for energy production following a long-term antiretroviral therapy.

The presentation by Bayurova Ekaterina (Chumakov Federal Scientific Center for Research and Developement of Immune- and Biological Products of Russian Academy of Science, Moscow, Russia) *Expression of HIV-1 Reverse Transcriptase in Tumor Cells of Epithelial Origin Modulates Mitochondrial Respiration and Motility* described that in mammary gland adenocarcinoma 4T1 cells, expression of HIV-1 reverse transcriptase (RT) led to an increased production of ROS, enhanced cell motility, and overexpression of mRNA of EMT factors including Twist, dependent on the levels of RT expression. In metabolic assays, RT-expressing 4T1 cells demonstrated enhanced basal respiration and spare respiratory capacity with no significant changes in glycolysis. Implanted into BALB/C mice, RT-expressing 4T1 cells caused faster tumor growth and higher metastatic activity than the parental 4T1 cells (p<0.05). Tumor size and metastatic activity were proportional to RT expression. On the contrary, in cervical cancer CaSki cells, expression of RT suppressed basal respiration decreasing spare respiratory capacity. Cells demonstrated a decreased expression of mRNA of Twist, Nrf2, and tubulins A and G, and reduced the directional cell motility, features consistent with the distruction of microtubuli. The results show that expression of HIV-1 RT modulates mitochondrial respiration differentially depending on the type of expressing cells, with the enhancement of respiration and cell motility in epithelial breast cancer cells, and suppression of respiratory capacity, basal cell respiration and cell motility in cervical cancer cells. Thus, HIV-1 RT appears to interfere into the interplay between respiration, mitochondrial morphology, microtubule and microfilament polymerization. Differential outcome of this interference may be due to differences in the functions of microtubuli and regulation of mitochondrial respiration in the metabolically rigid cervical cancer compared to metabolically plastic breast cancer cells. The latter corroborates recent findings on increased mitochondrial biogenesis and respiration in metastatic breast cancer [2].

Mikhail Golikov (Englehardt Institute of Molecular Biology, Moscow, Russia) in his presentation on *Metabolic Changes in Hepatocytes During HCV Infection: a Study in the Physiological Media* reported that cultivation of certain cell lines in physiologically based media such as Plasmax and HPLM, can increase their basal mitochondrial respiration and spare respiratory capacity. This effect is accompanied by the assembly of mitochondria into vast networks without changes in the mitochondrial mass. Noteworthy, in all cell lines tested, Plasmax significantly reduced lysosomal mass. Cells became much more sensitive to inhibitors of respiratory complex II and ATP synthase as well as to an inhibitor of fatty acid catabolism, pointing to an increased role of this pathway in the central carbon metabolism. Lower replication levels of various RNA viruses including HCV, influenza A virus and SARS-CoV-2 were observed in Plasmax medium compared to classic cell culture medium. Authors have registered that in Plasmax-cultured cells, all these viruses trigger oxidative stress. Furthermore, qPCR analysis in HCV-infected Huh7.5 cells identified changes in expression of various metabolic genes. The results strongly support that studies of metabolic changes in virus-infected cells, especially of those involving mitochondria, lysosomes, or redox systems, should be performed in physiological medium. Results presented by Golikov have been lately published in Antioxidants (Bazel) [3].

Marina Gottikh (Department of Chemistry and Belozersky Institute of Physicochemical Biology, Moscow State University) focused her Plenary Lecture on *Chronic Viral Infections and DNA Repair System of the Cell.* Viruses have developed various strategies to modulate DNA damage response (DDR), and some of them use DDR for their own benefit. This is the most interesting case as it gives us some new approaches to suppressing viral infection. Polyomaviruses induce and exploit the ATM and ATR signaling pathways. Inhibition of DDR kinases reduced the DNA replication of Merkel cell polyomavirus and BK polyomavirus. DDR is also involved in the life cycle of hepatitis B virus (HBV), chronically infecting hundreds of millions of people. The reason for the chronicity is a special form of the virus genome - circular covalently closed DNA (cccDNA), remaining insensitive to antiviral therapy. Inhibition of ATR and its downstream signaling factor CHK1, but not of ATM, decreased cccDNA formation during *de novo* HBV infection, and intracellular cccDNA amplification. Hepatitis C virus NS3 protein is shown to interact with DNA repair factors, Werner syndrome protein and Ku70, which is a component of DNA-PK, thereby diminishing the repair efficiency of the non-homologous end joining (NHEJ) pathway initiated by DNA-PK. HIV-1 can exploit the NHEJ pathway for its benefit. Integration of viral DNA into a host genome, performed by viral enzyme integrase, results in single-stranded gaps in the cell DNA that must be repaired. The lab of Dr Gottikh has analyzed the involvement of ATM, ATR and DNA-PK in the post‐integrational gap repair (PIR), and found that inhibition of DNA-PK and ATM, but not of ATR, significantly reduces PIR efficiency. This is a rather surprising result, given that both kinases are sensors for double-strand breaks that are not formed during integration. DNA-PK is recruited to integration sites due to binding to Ku70, and disruption of the binding disturbs HIV-1 replication. Using molecular docking experiments, inhibitors of the proteins’ interaction have been found and the leader compound is capable of suppressing PIR at micromolar concentrations. This discovery will open a new anti-HIV treatment based on PIR inhibition. Dr Gottikh concluded that elucidation of the interactions between viruses and DDR is important both for understanding the modulation of host cell functions by these pathogens and for developing new approaches to combating viral infections.

## Session 2: “Chronic viral infections and cancer”

The second session of the symposium included two sections, one on the mechanisms of viral oncogenicity, and the other, on viral infections, cancer and chronic inflammation.

The 1st section on **Mechanisms of viral oncogenicity** included the plenary lecture of Prof Maria Lina Tornesello, Istituto Tumori – IRCCS “Fondazione Pascale”, Napoli, Italy) on *Reversible and Irreversible Activation of Telomerases in HPV-Related Cancers* presented at symposium opening. The lecture highlighted the higher telomerase activity in most stages of HPV-related cancers, which is however functionally associated to the high levels of the viral E6 oncogene in the early stages of cancer progression, and later to the acquisition of mutations in the TERT promoter region, as preliminarily reported in HPV-related cancers [4] and in Hepathocellular Carcinoma (HCC) [5, 6]. These results indicate that promoter mutations have a much stronger effect on TERT activation in cervical neoplasia than the increased TERT expression associated with the HPV E6 oncoprotein. Moreover, this suggests that (i) Telomerase expression is reversibly regulated by viral E6 protein in the early stages of tumorigenesis, and (ii) Telomerase is irreversibly and highly activated by genetic alterations and promoter mutations in TERT gene in progressing cervical neoplasia. The identification of such two stages is critical for the molecular characterization of HPV-related cancers, along with their different prognosis and therapeutic responsivity. In particular, anti-HPV therapeutic vaccines could be highly effective within the first stage, while new actionable targets should be identified for SCC of the lower genital tract and in general for TERT mutated tumors.

Modulation of promoter activity underlying oncogenesis was also tackled in the presentation *Activity of Long Control Region of HPV as Determinant of Oncogenicity* by Prof Felicity Burt (Department of Medical Microbiology and Virology, University of the Free State, Bloemfontein, South Africa). Her data demonstrated that reporter gene activity downstream of an HPV6-derived LCR region with a 170bp duplication was significantly higher than the activity obtained with constructs made with LCR control containing no duplication. Similarly, enhanced transcriptional activity was observed for a reporter gene system constructed using HPV 31 derived LCR with nucleotide variations in the p97 promotor region. Enhanced transcriptional activity was observed with the mutant that possessed a single nucleotide change within the YY1 transcription binding site. Such data confirmed the knowledge that sequence variation within the LCR may have a functional effect on the activity of viral promotor, and that mutations in the non-coding region would represent potential biomarkers of an aggressive disease.

An oral presentation by Dudorova Alesja and Avdoshina Daria (Paul Stradins University Hopsital, and Riga Stradins University, Riga, Latvia; Chumakov Federal Scientific Center for Research and Developement of Immune- and Biological Products of Russian Academy of Science, Moscow, Russia) on *Comparative Characteristics of TC-1 and Novel 4TL-Based Cell Lines Expressing HPV 16 Oncoproteins E6 and E7 in Ability to Reproduce HPV-Associated Carcinogenesis in a Mouse Model* reported construction of adenocarcinoma 4T1 based cells expressing oncoproteins E6 and E7 of HPV 16, and comparison of the resulting subclones with a known model of HPV-associated cancer exploiting TC-1 cell line (https://www.atcc.org/products/crl-2493). TC-1 carry over 500, and 4T1luc2-based subclones only one copy of the E6/E7 genes. Nine 4T1 subclones were obtained that expressed different levels of E6/E7 mRNA. Interestingly, as in human cancer, expression of E6/E7 mRNA in 4T1luc2 induced expression of TERT. E6/E7-expressing 4T1luc2 demonstrated signs of G0/G1 arrest with diminished population of cells in S-phase. For TC-1, on the contrary, cells accumulated in the S-phase, the process correlated with expression of E7 mRNA. Both TC-1 and E6/E7-expressing 4T1luc2 subclones exhibited γ-H2AX foci indicating dsDNA damage, normally attributed to the production of ROS. Implanted into mice, both TC-1 and E6/E7-expressing 4T1luc2 cells formed solid tumors of similar size and growth rate (p>0.1). *Ex vivo* BLI demonstrated preferable infiltration of tumor cells into lungs in 4T1- and into spleen in TC-1 models, and similar infiltration into the liver, forming similar numbers of liver metastasis. Tumor size, organ infiltration by tumor cells, and number of metastasis were not correlated to the level of expression of E6/E7 mRNA. The latter observation corroborates the concept that expression of E6/E7 induces DNA damage that through accumulation of mutations allows HPV-driven cancers to become independent of the expression of viral oncogenes. This phenomenon was described in the plenary lecture presented on the Symposium by Prof Maria Lina Tornesello, and also demonstrated in the independent studies [7]. Overall, the parameters of tumor growth and metastatic activity of E6/E7-expressing 4Tluc2 cells were similar to that of TC-1. New subclones could be useful for testing HPV vaccines, as they allow to perform experiments in BALB/c mice syngenic to 4T1 cells. This model also allows to circumvent the drawback of high load of E6/E7 in TC-1 cells that may stimulate immune rejection of these cells in the E6/E7-immunized mice [8].

The oral presentation by Karen Kyuregyan (Russian Medical Academy of Continous Professional Education, and Peoples Friendship University, Moscow, Russia) on *Silent HDV Epidemics Culminates in High Levels of Liver Cirrhosis and Hepatocellular Carcinoma in the Population Despite 20 Years of HBV Vaccination* reported on the high frequency of HDV infection in Tuva. Bayesian analysis has shown that HBV had a long history of circulation in Tuva with the time to most common ancestor (MRCA) for predominant genotype HBV-D dating back over 1000 years. HDV circulation in Tuva started much later and was the consequence of two successive introductions of HDV genotype 1 (HDV-1). First wave was associated with HDV-1 sequences from the Central Asia and dates back to 1811 (95% HPD: 1741 – 1834). The second wave was associated with strains from Russia, its TMCA dates back to 1960s (95% HPD: 1953 – 1979). SkyGrid reconstruction of population dynamics showed an increase in the intensity of HDV spread since the 1990s peaking in 2010s. The reproduction number (Re) for HDV in Tuva calculated based on the population dynamics predicted using Birth-Death Skyline analysis increased rapidly after 2010s, reaching the plateau of about 3 cases of infection from one source in recent years. This group also observed the rise in predicted Re values for HBV in Tuva, from less than 1 before 2000s to 5 after year 2000. At the same time, a serosurvey of healthy volunteers has shown the average detection rate of HBsAg with anti-HDV to be 1.0%, which was significantly lower compared to data from a similar serosurvey done in 2008 (2.3%, p = 0.0218). Importantly, a serosurvey done in 2019 detected no anti-HDV positive samples among participants under 30 years. HBsAg/anti-HDV positivity rate peaked at 7.4% in the age group of 50-59 years, prevalence of HBV/HDV in this population group has increased since 2008 five-fold. Authors associate the increase in the intensity of the spread of HDV in Tuva with HBV circulation in non-vaccinated adults, with a large number of people living with HBV who are susceptible to HDV superinfection. HDV epidemics revealed by Kyuregyan K et al. explain the high rates of liver cirrhosis and hepatocellular carcinoma observed in Tuva. These data demonstrate that a massive HBV vaccination program for newborns does not limit the spread of HBV and HDV infections in populations dominated by non-vaccinated middle-age and elderly people and urges for implementation of massive HBV vaccination, HDV screening and chemo- and immunotherapy of HBV-infected aimed at HBV eradication as the healthcare policy for endemic regions.

Toyé Rayana (Cancer Research Center of Lyon, INSERM, CNRS, Lyon, France) in her short presentation on *MicroRNAs Profiling in Senegalese HBV-Infected Patients* reported profiling, using NanoString’s nCounter® technology, of approximately 800 circulating miRNAs in a retrospective cohort of 34 Senegalese patients, 17 with chronic hepatitis B (CHB) and 17 with HBV-related hepatocellular carcinomas (HCCs). MiRNA are small, non-coding single-stranded RNAs that can modulate target gene expression at the post-transcriptional level. Regulation occurs mainly by binding to complementary sequences in the 3′-untranslated region (UTR) of target mRNAs and then integrating into RNA-induced silencing complexes to suppress translation or to degrade miRNA-bound mRNA transcripts [9]. A total of 404 miRNAs were detected, 58 miRNAs were quantified in at least 75% of patients. Principal component analysis (PCA) detected no differences in representation of circulating miRNAs with age, viral load, HBe status or clinical parameters such as ALT, AST. At the same time, PCA revealed differential expression of nine miRNAs (let-7g-5p, miR-122-5p, miR-181a-3p, miR-210-3p, miR-2682-5p, miR-300, miR-451a, miR-514a-3p and miR-519c-3p) in the HBV-related HCCs as compared to CHB (*p *< 0.05). Six of them (let-7g-5p, miR-122-5p, miR-210-3p, miR-300, miR-451a and miR-519c-3p) shared a network of regulatory pathways and target genes (e.g MYC, BCL2L1, HIF1A, and BMI1) involved in the control of cell proliferation and immune system. In particular, miR-122-5p, reported to participate in the regulation of various cancers, was strongly downregulated in HCC confirming its onco-suppressor role in HCC [10]. Finding pattern(s) of modified expression of miRNA characteristic to HBV-related HCC has important clinical implications. Such miRNAs, in particular, miR-122-5p, could be used as candidate biomarkers in a composite clinical diagnosis/prognostic score. The authors plan to evaluate the potential of differentially expressed circulating miRNAs they detected in HBV-related HCCs to predict development of HCC in a larger, longitudinal followed cohort of CHB patients.

The 2nd section on **Viral infections, cancer and chronic inflammation** started with the Plenary Lecture of Nicolas Noel (MCU-PH, Service de Médecine Interne et Immunologie Clinique, GHU Paris Saclay, AP-HP BICÊTRE/CEA 1184, Paris, France) on *Chronic Inflammation in HIV-1 Infection, are Elite Controllers Different?* Antiretroviral therapy (ART) of human immunodeficiency virus (HIV) infection is usually mandatory to maintain an undetectable viremia and to preserve immune functions. There are, however, HIV-1 elite controllers who harbor replication competent virus, but are able to control its replication without antiretroviral therapy. A hallmark of HIV-induced pathology is immune activation occurring already at the early stages of infection. In his plenary lecture, Dr Noel described multiple sources of immune activation such as: viral replication and release of virions, antigen presentation, microbial translocation of mucosal origin, activation of the interferon pathway, viral co-infections and others. Immune activation is also linked to the control of the HIV reservoir and viral latency, as well as to the risk of clinical events such as cardiovascular or neurocognitive diseases. ART reduces chronic immune activation, but does not necessarily normalize the inflammatory parameters. HIV-1 controllers allow to study the causes and consequences of inflammation. Plenary lecture provided the evidence of persistent immune activation in HIV-controller patients in comparison with controlled patients under ART, and discussed the risk of viral evolution and the research perspectives in this field.

Plenary lecture on chronic inflammation in HIV infection was followed by the oral presentation by Benoit Favier (DRF-IBFJ-IDMIT, and CEA-University Paris Saclay-Inserm, Fontenay-aux-Roses, France) on the *Dynamics of LILRB2 Immune-Checkpoint in HIV and SIV Infections*. Dendritic cells (DCs) play an important role in initiating and regulating adaptive immune responses leading to the control of viral infections. However, HIV infection dysregulates DC functions which may in-part account for viral persistence. Studies by Favier et al. indicate that this dysregulation is induced by the interaction of the inhibitory receptor LILRB2 (leukocyte immunoglobulin-like receptor subfamily B member 2) which acts to suppress the immune system with its MHC-I ligands. Inhibitory receptors, and paired activating receptor siblings, are critical regulators of innate immunity and inflammation [11]. In HIV and SIV infections, interaction of LILRB2 with MHC-I is associated with the rate of progression of the disease. Dr Favier presented the dynamics of the LILRB2/MHC-I inhibitory axis in DCs during different phases of the infection. Primary phase of HIV infection was characterized by a strong increase of LILRB2 and MHC-I expression on the surface of DCs. The dynamics of LILRB2 and MHC-I in the early phase of infection in blood and tissues was further characterized in a macaque model of SIV infection. The study revealed an up-regulation of LILRB2 and MHC-I not only on DCs, but also on macrophages, starting from the first week after the onset of SIV infection. These results identify LILRB2 as a potential target to improve DC functions and thus anti-viral adaptive immune responses in early stage of retroviral infection. The lecture presented the latest tools to assess the in vivo role of LILRB2. In conclusion, Favier et al. proposed LILRB2 as a target of immunotherapy in HIV infection. Interestingly, in endometrial cancer, knockdown of LILRB2 results in a dramatic decrease in the proliferation, colony formation and migration of cancer cells, and in a notable reduction of tumor growth in in vivo xenograft experiments [12] which suggests analogies between directions to cancer and HIV infection cure.

Presentation of Sara Svensson Akusjärvi (Karolinska Institutet, Stockholm, Sweden) on *The CD4*+*CCr6*+ *T Cell Compartment is Unique in EC Compared to Long-Term ART-Treated Individuals* focused on the unique profile of CCR2 and CCR6 in lymphocyte populations of people living with HIV who are elite controllers of HIV infection (PLWHec) as compared to PLWH on ART (PLWHart) and healthy controls (HC)*.* Flow cytometry analysis identified a significant decrease of CCR6 and CCR2 on CD4^+^ and CD8^+^ T cells in PLWH_EC_ compared to PLWH_ART_ as well as to HC for both CD4+ and CD8+ T cells. In PLWHart, CCR2 on CD8+ T cells was reduced also if compared to HC. From the sorted cell populations, CD4^+^T cells of PLWHec expressed an enrichment of interferon-α response, mitochondrial oxidative phosphorylation (OXPHOS) and decreased glycolysis as compared to PLWHart (all *p* values < 0.05). Specifically, CD4^+^CCR6^+^ cells demonstrated an enrichment of apoptosis and p53 signaling in PLWHec compared to PLWHart (adj *p <* 0.05). The phenomenon was not seen in CD4^+^CCR6^-^ cells. On the overall, flow cytometry analysis revealed a unique expression profile of CCR2 and CCR6 in PLWHec, while the profile in PLWHart was similar to HC. Interestingly, normal cells are characterized by enriched OXPHOS and decreased glycolysis, while in various cancer cells glycolysis is enhanced and OXPHOS capacity is reduced [13]. Furthermore, the authors observed an enrichment of apoptosis and p53 signaling in CD4^+^CCR6^+^ cells from PLWHec showing susceptibility to cell death by apoptosis, whereas primary mode of cell death in HIV-1 infected T cells does not involve apoptosis [14]. CD4^+^CCR6^+^ T cells have been proposed as highly permissive to HIV-1 and major contributors to the viral reservoir [15]. Svensson S. et al*.* hypothesized that the reduced frequency of CD4^+^CCR6^+^ cells, their susceptibility to cell death by apoptosis and metabolic profile with increased OXPHOS and decreased glycolysis could potentially aid in achieving natural control of HIV-1.

Sona Chowdhury (University of California, San Francisco, USA) focused her presentation on *Programmed Cell Death Protein 1 (Pd-1) and Programmed Cell Death Ligand 1 (Pd-L1) Expression Profile by Immune and Epithelial Cells in Primary and Metastatic Cervical Cancer Tissue*. PD-L1 was expressed in epithelial cells of 70% primary squamous cell carcinomas (SCC, n=12), while 30% were negative. PD-L1(+)-SCC samples revealed heterogeneous PD-L1 expression patterns in-between tumors and within each tumor, mostly seen as membranous focal staining, both in the periphery and in the center of invading nests, and also at the tumor-stroma interface. Similar heterogeneous staining was characteristic to proximal lymph nodes (LNs, n=8) affected by metastasis. Stroma of nearly 50% of PD-L1(+)-SCC was rich in PD-L1 positive immune cells (ICs). PD-L1 expression in ICs in the stroma was observed also in tumors negative for PD-L1. Besides, PD-L1(+)-ICs were observed also in the stroma of 50% of metastatic LN samples. Stromal ICs also expressed PD-1. No PD-1 expression was observed in the epithelial cells in either SCC, or HSIL, or LN tissues. Authors semi-quantified the number of PD-1(+) ICs. Over 70% of primary SCC had moderate to high number of PD-1(+) ICs in the stroma. PD-1(+) ICs were observed also in HSIL samples. In the stroma of LNs, levels of PD-1 expression in IC were lower. Levels of PD-1 expression by IC in SCC and stroma were not correlated to the expression of PD-L1 by the epithelial cells. The manual IHC scoring was validated by a bioimage analysis software. In conclusion, varied PD-L1 expression patterns were observed both in primary and metastatic SCC. PD-L1 expression was seen not only in epithelial cells but also in ICs. In contrast, PD-1 expression was restricted to ICs. Level of PD-L1 expression varied in-between SCC samples and within each of the samples. This heterogeneous pattern of PD-L1/PD-1 expression may contribute to the immune-mediated pathogenesis of CC and warrants further investigation.

Ivan Trotsenko (Peoples Friendship University of Russia, Moscow, Russia) focused his presentation on *Violaton of Gene Regulation in Colon Cancer (CC) and Adjacent Colon Mucosa Revealed by Comparative Gene Expression and Gene Correlation Analysis*. Tissue samples from tumor (T) adjacent mucosa (AM) and normal control (NC) were analyzed for gene expression levels and gene correlation coefficients. Principal component analysis (PCA) of expression profile revealed significant activation of proliferative, matrix metalloproteinases, antiapoptotic and angiogenic pathways in tumor compared to only slight difference observed between NC and AM. On the PCA plot T samples were separated from NC and AM samples and their gene expression evaluated. Correlation of gene expression in NC differed significantly from that in T and AM samples, whereas the latter two groups did not differ. Cluster analysis revealed that correlation scores of genes in NC were significantly higher than correlation scores in T and AM, i.e. gene expression patterns in normal control tissues, but not in T or AM, were strongly coordinated. To conclude, analysis of gene expression profile in CC samples demonstrated high level of proliferative activity, angiogenesis and apoptosis inhibition. Although gene expression patterns and the level of expression of cancer hallmarks in mucosal tissues surrounding the tumors and normal colon epithelium did not differ, interaction of these pathways in adjacent mucosa was as disrupted as in tumors. Thus, gene correlations analysis revealed violations of gene interaction not only in the tumor, but also in the surrounding morphologically unchanged epithelium.

The first day of the Symposium was concluded with the Plenary Lecture of Margaret Liu (Protherimmune, and University of California, San Francisco, USA) on *Nucleic Acid Vaccines and their Potential for Improving Non-Specific Immune Responses and Immune Fitness for Chronic Infections and Cancer*. Immunotherapies for chronic viral infections and cancer have utilized approaches including monoclonal and bispecific antibodies, immunodulatory agents (cytokines and checkpoint inhibitors), vaccines (utilizing various delivery systems), and adoptive cell therapies. The efficacy of certain antibodies, immunomodulators, and cell therapies for certain cancers have demonstrated their potential. This has led to increased efforts to develop immunotherapeutic vaccines targeting tumor and viral antigens. But challenges remain even after selecting an appropriate antigen to target, whether a viral protein or a tumor antigen. One reason for this is that tumors and chronic infections can affect the immunological milieu resulting in tolerance or immune suppression. The lecture by Margaret Liu described the use of nucleic acid vaccines as potential vectors for therapeutic vaccines for both chronic infections and cancer with the focus on the immune mechanisms stimulated by these vaccines, including innate immunity and how these innate immune responses may contribute to the specific immune responses against an encoded antigen and what non-specific immune benefits they can give including immune fitness.

The second day of the Meeting was opened with the Plenary In-Sight Lecture of Fabien Zoulim (Hepatology Department, Hospices Civils de Lyon; Cancer Research Center of Lyon, Lyon, France) on *The Path Towards the Cure of Chronic HBV Infection*. Despite the implementation of universal vaccination programs, chronic HBV infection remains a major public health problem worldwide. Moreover, existing therapeutic compounds against HBV are limited and mainly include nucleos(t)ide analogues (NUCs) (i.e. entecavir, tenofovir) and pegylated interferon α (Peg-IFN-α). As beneficial as they may be, these treatments do not usually achieve eradication of the virus and HBsAg loss is still rare. Thus, these regimens require indefinite treatment to maintain viral suppression and prevent the virological relapse that usually occurs after treatment discontinuation. Moreover, it is unrealistic to expect all patients to adhere to long-term or lifelong non-curative treatment and there is a strong patient preference for therapy termination. Drug resistance is still a concern in low-income settings that use early generation NUCs and while there is no resistance with IFN treatment, the use of this agent is rare because of problems with tolerability. The cost of lifelong therapy and monitoring is also an important economic issue in highly endemic areas. Thus, the aim of new therapeutic strategies is to achieve a “functional cure” for chronic hepatitis B (CHB), defined as sustained off-treatment loss of HBsAg, undetectable HBV DNA in serum, normalization of liver enzymes and improvement in liver histology. HBsAg loss is a sign of profound suppression of HBV replication and is the only existing indicator for safe treatment discontinuation. Moreover, HBsAg loss is associated with a decreased risk of developing inflammation-driven hepatic complications such as HCC. The lecture highlighted the current and the potential anti-HBV advanced strategies to be developed in the next years. In particular, Prof Zoulim described the novel direct-acting antivirals against HBV infection articulated in a) DAA targeting the HBV replicative cycle (including entry inhibitors, capsid assembly modulators, HBsAg secretion inhibitors and Nucleos(t)ide reverse transcriptase inhibitors) and b) DAA targeting HBV gene expression (i.e. small-interfering RNAs-siRNAs and antisense oligonucleotides -ASOs). In parallel novel host-targeting agents against HBV infection have been developed to stimulate a) the innate immune response (in hepatocytes via retinoic acid-inducible gene I (RIG-I) or in neighboring cells via Toll-like receptor (TLR) and/or b) adaptive immune response (with checkpoint inhibitors or therapeutic vaccines). Finally, combinations of such treatments have been reported (DAA combinations with or without inhibition of HBV expression or with immunotherapy [16].

In order to meet the 2030 deadline for the elimination of viral hepatitis, several further challenges must be addressed. In particular, the development of better animal models for the study of HBV infection and antiviral drug discovery is needed. These models could help overcome the challenges of HBV cccDNA targeting, evaluating immune stimulation and preclinical testing of drug combinations. Innovative therapeutic strategies are on the way as the chimeric antigen receptor (CAR) T cells, HBV T-cell receptor (TCR)-designed CD8 T cells and soluble TCRs, to redirect HBV-specific T cells to infected hepatocytes and gene editing approaches to directly target cccDNA by clustered regularly interspaced short palindromic repeat (CRISPR)/Cas9-based approaches. In summary, HBV eradication will require a thorough understanding of HBV biology, the specificities of the liver microenvironment and their interactions with the immune system. The design of future therapeutic approaches against HBV will need to take these factors into account, as they will probably pave the way for the next generation of antiviral agents and their combinations.

## Poster session 3

Plenary in-sight lecture was followed by the first session of the second day dedicated to the poster presentations. Posters were presented in the form of short (5 min) orals. The first poster was by Amie Ceesay (Cancer Research Center of Lyon, INSERM, CNRS, Lyon, France) who reported on the *Concordance of the Xpert Hepatitis B Viral Load Test and Conventional Quantitative PCR in Detecting and Quantifying Viremia Using Stored Plasma and Dried Blood Spot Samples in West Africa.* Ceesay et al. analyzed 266 stored plasma samples from patients at various hepatitis B disease stages. Of these, HBV viral load results were concordant between the Xpert and the conventional qPCR: 48.9% were detectable, and 23.7% undetectable by both assays. At the same time, the results were discordant in 26.7% samples that were detectable by Xpert but not qPCR, only two samples were detectable by qPCR but not Xpert. Quantities of HBV DNA determined by Xpert and qPCR were in good agreement. At the same time, significant bias was observed between the results of Xpert assay of plasma and dried blood samples (DBS), towards lower viral loads in DBS. Bias was smaller at lower viral loads. In conclusion the Xpert HBV DNA test provided a reliable alternative for detecting and quantifying HBV DNA viral load using stored plasma samples, but not in the reconstituted dried blood samples.

In the second poster, Baranovskii Denis (Research and Educational Resource Center for Cellular Technologies, Peoples’ Friendship University of Russia, Moscow, Russia and Department of Regenerative technologies and Biofabrication, National Medical Research Radiological Center, Obninsk, Russia) presented data on *Early Hypocoagulation Predicts Severe Acute Respiratory Distress Syndrome in Unvaccinated Patients with Covid-19 Related Pneumonia.* Microcirculation abnormalities associated with coagulopathy may lead to acute respiratory distress syndrome (ARDS) in COVID-19 patients. In order to identify parameters that may early indicate further respiratory failure or severe ARDS requiring treatment in an intensive care unit (ICU), Baranovskii et al analyzed the differences in prothrombin time and international normalized ratio (INR) as well as in Fibrinogen and D-dimer levels between unvaccinated COVID-19 patients who had been transferred to an ICU within two weeks after admission and matching cohort of COVID-19 patients with stable course of the disease. Multiple comparisons showed statistically significant prolonged prothrombin time on admission in ICU-transferred COVID-19 patients compared to the stable COVID-19 patients (14.15 compared to 13.25 sec, respectively; p-value=0.0005), whereas other parameters, such as levels of D-dimer, fibrinogen and INR levels did not differ. In conclusion, appearance of an incipient hypocoagulation appeared as an early indication for the development of severe complications of COVID-19 requiring transfer to ICU.

The third poster by Bayurova Ekaterina et al (Gamaleya Research Center for Epidemiology and Microbiology, Moscow, Russia) described the *Enhanced Motility of Adenocarcinoma Cells Expressing HIV-1 Protease.* High motility is an important factor determining metastatic activity of cancer cells. In HIV-1 infection, high capacity for migration and trafficking contributes to efficient spread of infected cells. Bayurova E et al studied the effects on motility of cancer cells of HIV-1 protease (PR). PR was inactivated by mutagenesis to make it less toxic and through this to increase levels of eukaryotic expression, however, after inactivation, the enzyme retained up-to 30% of the activity. Interestingly, expression of HIV-1 PR caused enhanced motility in vitro and increased metastatic activity in vivo, specifically for tumor cells with high level of PR expression. The motility was not associated with oxidative stress, or expression of markers of epithelial mesenchymal transition (EMT) as their expression in PR-expressing tumor cells were the same as in the parental cell line. On the contrary, treatment with antioxidants led to an increase of motility, at least in vitro. Authors hypothesized that increased motility of PR expressing tumor cells could be due to residual enzymatic activity of PR. In tumor cells, protease treatment disrupts cell-to-cell contacts and promotes cell migration, the mechanism totally unrelated to EMT. Treatment with antioxidants promotes the process by rescuing protease activity via cleavage of disulfide bonds. In conclusion, HIV-1 protease induced an increased cell migration (motility) in vitro and in vivo not associated with the induction of oxidative stress and not consistently associated with the expression of the EMT proteins, i.e. independently of EMT, but due to intrinsic HIV-1 PR activity. This phenomenon may stand behind high motility of HIV-1 infected cells that contributes to efficient viral spread in the infected subjects.

In the fourth poster, Gordeychuk Ilya et al. (Chumakov Federal Scientific Center for Research and Developement of Immune- and Biological Products of Russian Academy of Science, Moscow, Russia) described *Culturing and Phenotypic Characterization of Primary Hepatocytes of Common Marmosets (Callithrix Jacchus) as a Platform to Create Cell Lines Expressing Viral Proteins and Human Tumor-Associated Antigens.* Primary hepatocytes of Common Marmoset (CM) have been reproducibly obtained and cultured up to one month without detectable changes in either cell phenotype, or transcription of hepatocytes specific genes. Albumin secretion level was 47 μg/mL. After one month, cells underwent spontaneous phenotype change and were cultured for the following six months without signs of cell crisis. Hepatocytes were transduced by lentivirus encoding telomerase reverse transcriptase (TERT). TERT-transduced hepatocytes differed from parental cells in the increased TERT activity evaluated by TRAP assay. Expression of TERT led to stabilization and shortage of doubling time but did not promote either colony formation or collagen-independent growth characteristic to completely transformed (malignant) cells. In conclusion, primary hepatocytes of the common marmoset can be stably cultured for at least one month, transduced with lentiviruses to express tumor associated antigens as TERT, and represent a platform to generate immortalized cell lines.

The fifth poster by Sharabrin Sergei (State Research Center of Virology and Biotechnology “Vector”, Koltzovo, Novosibirsky Region, Russia) described *Obtaining of Influenza mRNA-Vaccines Encoding Artificial Antigens Using Modified Analogs of Nucleotides.* mRNA vaccines are a relatively new vaccine platform that has been adopted to develop therapeutic and immunoprophylactic vaccines against oncological and infectious diseases. The use of mRNA vaccine technology makes it possible to rapidly construct vaccines against actual circulating influenza virus strains. In the current study for developing mRNA-based vaccine constructs, an alternative approach aimed at designing a universal influenza virus vaccine by encoding two variants of the influenza hemagglutinin stem (i.e., AgH1 and AgH3) and a conserved M2 protein (AgM2), has been used. Previous studies demonstrated that immunization of BALB/c mice with a combination of DNA vaccines encoding those antigens evoked both humoral and cellular responses, as well as a moderate statistically significant cross-protective effect against two heterologous viruses: A/California/4/2009 (H1N1pdm09) and A/Aichi/2/68 (H3N2). The current results reveal that modified naked mRNA vaccines encoding artificial antigens and constructed with conserved fragments of the hemagglutinin stem of influenza viruses, H1N1 and H3N2, as well as the M2 protein, can induce a specific antibody response against influenza virus in mice

In the sixth poster, Rudometov Andrey (State Research Center of Virology and Biotechnology “Vector”, Koltzovo, Novosibirsky Region, Russia) reported the *Design of Polyepitope HIV Immunogens Including Linear Epitopes Recognized by Broadly Neutralizing Antibodies.* The study aimed to develop a mRNA-based anti-HIV vaccine encoding synthetic proteins composed of panels of epitopes for HIV neutralizing antibodies (polyepitopes). Three variants were constructed, their genes were optimized for expression in eukaryotic cells. Resulting sequence were cloned into a vector, under the T7 promoter, open reading frame flanked by 5' and 3' untranslated regions, and respective mRNAs were generated. Variant 6.1.2 encoded soluble, and variants 9.2.5 and 10.1, insoluble proteins. Their structure was characterized by circular dichroism spectroscopy. Presence of epitopes of antibodies neutralizing HIV-1 was confirmed by immunochemical analysis. Variant 6.1.2 encoding soluble polyepitope was recloned into eukaryotic expression vector pVax1 to be used as a template to produce a prototype HIV-1 mRNA vaccine. Further studies are planned to characterize the immunogenicity of the polyepitope encoding mRNA and its ability to induce the production of neutralizing antibodies.

Next poster presented by Kostyushev Dmitry (National Medical Research Center of Tuberculosis and Infectious Diseases, Moscow, and Sirius University of Science and Technology, Sochi, Russia) described *Tools and Technologies for Inducible and Reversible Packaging of Crispr/Cas9 Ribonucleoprotein Complexes and Their Targeted Delivery by Biological Nanoparticles.* Light-inducible dimerization system has been developed genetically fusing Streptococcus thermophilus StCas9 protein (CRISPR locus 1) with a light-inducible dimerization domain and RNA hairpin-interaction domain. A second partner of light-inducible dimerization system was fused with an intraluminal part of a constitutive protein of biological nanoparticles. An array of HBV-targeting sgRNA with RNA hairpins was generated using genetic engineering and enabled tunable packaging of Cas9 protein with sgRNA. CRISPR/Cas9-loaded nanoparticles significantly reduced HBV replication intermediates in vitro. Functionalization of generated nanoparticles with variants of CD63 protein fused to liver-enriched peptide was envisaged to achieve tissue-specific delivery of CRISPR/Cas9 RNPs; a 7-fold increase in uptake by hepatocytes has been demonstrated in HepG2 cells. In conclusion, Kostyshev D et al developed a system for simultaneous, inducible and reversible packaging of CRISPR/Cas9 RNPs and demonstrated its potent antiviral activity against HBV in vitro.

Poster session was concluded by Jansons Juris (Latvian Biomedical Research and Study Center, Riga, Latvia) with the poster on the *Efficacy of Co-Transfection and Levels of Co-Expression of Two Fluorescent Reporter Proteins in Co-Transfected Cells.* The study was performed to optimize and standardize the process of co-delivery of “reporter” plasmid plus “test” plasmid needed to evaluate the effects of the protein encoded by the “test” gene. In optimization experiments, both delivered plasmids were encoding reporters: one expressed green fluorescence protein (GFP, pVaxGFP) and the other, near-infrared protein iRFP670 (pVaxiRFP670). Plasmids, in varying amounts and varying ratios, were delivered with the help of transfection reagent Turbofect^TM^ (Thermo Fisher Scientific). Results of co-expression were assessed by flow cytometry evaluating cells expressing GFP, or iRFP670 and both reporters. Co-delivery experiments demonstrated that irrespectively of the plasmid amount and ratio, nearly 90% of transfected cells express both reporters. Interestingly, also, iRFP670 was found to be toxic to expressing cells, as was registered by reduction of the number of viable cells after transfection. Also, increase in the ratio of pVaxiRFP670 in the plasmid mixture increased the level of expression of iRFP670, but deceased the expression of GFP, not affecting the percent of co-expressing cells. It may relate to extreme stability of iRFP670 [17], which may result in induction of ER stress, namely unfolded protein response which halts protein synthesis [18]. Results presented by Jansons J et al*.* demonstrate that it is necessary to keep in mind that test protein can negatively affect the metabolism of the target cell causing an unspecific loss of the reporter signal, unrelated to the activity to be measured by reporter assay, resulting in incorrect interpretation of test results.

## Session 4: “Immune response in chronic viral infections and cancer”

Fourth session of the symposium was devoted to the immune response in chronic viral infections and cancer and contained two sections. The first on **Innate and adaptive immunity weak points** was opened by the Plenary Lecture of Julia Kzhyshkowska (Institute of Transfusion Medicine and Immunology, Medical Faculty Mannheim, Heidelberg University, Mannheim, Germany) on *Macrophages as Viral Targets and Reservoirs for Long-Term Survival.* Viruses manipulate cell biology to utilize monocytes/macrophages as vessels for dissemination, long-term persistence within tissues and virus replication. Viruses enter cells through endocytosis, phagocytosis, macropinocytosis or membrane fusion. These processes play important roles in the mechanisms contributing to the pathogenesis of these agents and in establishing viral genome persistence and latency. Upon viral infection, monocytes respond with an elevated expression of proinflammatory signaling molecules and antiviral responses, as is shown in the case of the influenza, Chikungunya, human herpes and Zika viruses. Human immunodeficiency virus initiates acute inflammation on site during the early stages of infection but there is a shift of M1 to M2 at the later stages of infection. Cytomegalovirus creates a balance between pro- and anti-inflammatory processes by inducing a specific phenotype within the M1/M2 continuum. The majority of viruses employ monocytes/macrophages as a repository but certain viruses also use these cells for productive replication. Infected macrophages generally display abolished apoptosis and restricted cytopathic effect, sustaining virus production and facilitating inflammation. In conclusion, viruses adapt their behavior to enter monocytes/macrophages, develop strategies for the immune escape, reprogram infected macrophages and manipulate their activity.

In the following oral, Jorma Hinkula (Linköping University, Linköping, Sweden) described the *Impairment of Nk-Dc Cross-Talk as a Component of Immune Dysfunction in HIV-1 Infection.* The study was focused on the impact of different forms of HIV-1 (free virion, bound to complement factors or to immune-complex) on antigen-presentation and immune activation of DC cells. Immature dendritic cells exposed to free HIV-1 remain capable of supporting enhanced NK cell antiviral cell-signaling and activity. Instead, presentation of HIV-1 complexed with complement factors or immunoglobulin complexes prior to uptake and presentation by DCs, result in suboptimal and considerably more limited antiviral activity of NK cells. One major condition to provide an immune reaction suppression in NK cells seem to be the cell-to-cell contact between the NK-and-DC cells exposed to complexed HIV-1 forms. The lecture offered different underlying mechanisms of how these phenomena may occur.

Nosik Marina & Petrakova Natalia (Mechnikov Serum Institute, and Gamaleya Research Center for Epidemiology and Microbiology, Moscow, Russia) spoke on the *Impaired Production of Pro-Inflammatory Cytokines in HIV-Infection Associated with Tuberculosis.* Atypical course of TB in the late stages of HIV-infection leads to the late detection of *M. tuberculosis* infection/delayed diagnosis of TB, resulting in HIV-1/TB co-infected patients not receiving the TB treatment with prolonged circulation of *M. tuberculosis*. Circulation of *M. tuberculosis* in HIV-1 patients has profound effects on the immune system, it negatively regulates T-cell activation while activating HIV-1 replication. Specifically, co-infection deregulates the production of cytokines necessary to control both infections, aggravating disease progression and compromising the effects of ART and TB treatment. Study aimed to characterize the expression of pro- and anti-inflammatory cytokines in patients with HIV- or TB mono-infections and HIV/TB co-infection before and during antiretroviral (ART)/anti-tuberculosis therapy (ATT), and to identify predictive markers of treatment efficacy, morbidity and mortality. IL-6 is a major factor in chronic inflammatory diseases and high level of IL-6 is an early marker of *M. tuberculosis* infection. The other pro-inflammatory cytokine IL-8 activates leukocytes, specifically neutrophils, and causes their chemotaxis into the focus of inflammation. Hypersecretion of IL-8 accelerates this process and enhances local inflammation and tissue damage, contributing to the pathogenesis of HIV/TB-co-infection. Besides, elevated IL-8 levels manifest the development of immune reconstitution inflammatory syndrome (IRIS); IRIS on the background of TB associates with poor prognosis and mortality, confirmed in this study. Authors presented data on the correlation of IL-6 hypersecretion in HIV patients with TB-infection, and of hypersecretion of IL-6 and IL-8 with an aggravated course of HIV/TB co-infection characterized by a severe lung damage. The effects were analogous to the role of IL-6 and IL-8 in development of lung lesions in the acute stage of SARS-CoV-2, as well as in the pathogenesis of lung cancer. Authors also reported the high levels of IL-18 associated with ART/ATT failure, suggesting it as a potential marker to monitor the efficacy of treatment. Besides, high levels of IL-18 contribute to chronic low-grade inflammation, and are associated with an increased risk of cardiovascular complications**,** metabolic disorders and non-AIDS associated cancers, indicating that high levels of IL-18 can serve to predict the development of HIV-associated pathologies. At the same time, researchers noted the reduced secretion of TNF-α and IL-17 resulting in inability of the body to form granulomas thus promoting *M. tuberculosis* spread and development of generalized TB. The latter observation was confirmed by correlation of IL-17 levels with the severity of TB forms. IL-17 also acts as tumor suppressor during the process of tumorigenesis by enhancing NK cell and CTL cell activation, and recruiting neutrophil, NK cell, CD4+ and CD8+ T cell infiltration into tumor tissues, hence, reduced levels of IL17 may favor the development of cancer lesions. Authors plan to investigate if IL-6, IL-8 and IL-18 hypersecretion and reduced secretion of IL-17 in HIV, TB mono-infections and HIV/TB co-infections can predict morbidity and mortality associated with lung and other forms of cancer.

Anna Lucia Tornesello & Franco Maria Buonaguro (Istituto Nazionale Tumori – IRCCS Fondazione Pascale, Naples, Italy) presented data on *Profiling the HCV Immune Response in Patients with HCV-Related Diseases by Peptide Microarray Analysis*. In order to identify new biomarkers specific for progression to cancer, sera from HCV-infected patients have been characterized. In particular plasma samples of HCV positive subjects, including HCC patients and cryoglobulinaemic patients as well as asymptomatic HCV chronic infected subjects have been analyzed on a peptide microarray platform made of 5952 overlapping 15-mer synthetic peptides covering the whole HCV proteome (C, E1, E2, NS2, NS3, NS4A, NS4B, NS5A, NS5B and P7). Overall, the level of anti-HCV antibodies was significantly higher in HCV-positive cryoglobulinaemic subjects versus all other groups of HCV-positive patients. Particularly, a statistically significant stronger immune response was observed against the C-terminal peptides of the C protein in cryoglobulinaemia *versus* HCV chronic infected subjects (*P*<1*10^-6^). Both HCC and MC showed a significant stronger immune response against peptides of C, E2, NS3, NS4A, NS4B, NS5A and p7 compared to HCV-positive subjects with chronic hepatitis, indicating their possible use as new biomarkers for HCV-related neoplasia among chronically HCV infected subjects.

Isaguliants Maria (Riga Stradins University, Riga, Latvia) in her presentation on *Enzymatic Activity of RNA Dependent RNA Polymerase in Chronic Hepatitis C Modulates the Induction of Type I Interferons* questioned if abnormal (ABN) or persistently normal liver enzyme levels (PNAT) in chronic hepatitis C are linked to the enzymatic activity of RNA dependent RNA Polymerase (RdRps) of HCV. For this end, authors sequenced RdRp of 17 PNAT and 25 ABN patients. No difference was found in either consensus sequences nor covariance networks in these selections. Next, coding sequences for RdRp variants from PNAT (n=9) and ABN patients (n=8) were cloned and expressed in HEK293 cells, and their capacity to drive reporter expression from the internal ribosome entry site of HCV (HCV IRES), was assessed by Prof Lee JC et al (Kaohsiung Medical University, Taiwan) in a previously described model [19]. RdRps from PNAT and ABN patients did not differ in the enzymatic activity exhibited on HCV IRES. At the same time, specific activity was positively correlated with HCV viral load, indicating that RdRp activity is not a factor behind liver function. Interestingly, however, RdRp activity correlated with high significance to the level of fibrosis, but not to the other parameters of Knodell Score, i.e. was strongly associated with inflammation. Authors have also evaluated the capacity of RdRps to perform synthesis of short dsRNA in the stage of initiation of polymerization. This was achieved in vitro by co-transfecting HEK293 cells with plasmid encoding RdRp and plasmid encoding luciferase (Luc) under the control of IFN-β promoter. The IFN-β promoter is activated by dsRNA via RIG-1-regulated pathway. Again, as in case of specific enzymatic activity, the effects on IFN-β promoter of RdRps derived from PNAT and from ABN patients did not differ. Interestingly, the capacity to activate IFN-β promoter was inversely correlated to specific RdRp activity. RdRp variants with high specific activity caused 2-3 fold inhibition of IFN-β promoter driven expression of Luc. Authors explained it by less active RdRps unable to efficiently synthesize the full-length Luc mRNA, produce instead an excess of short dsRNA resulting from repetitive initiation of RNA synthesis sensed by RIG-I. The phenomenon was not related to the origins of RdRps (whether they were derived from patients with PNAT or ABN status); RdRps derived from ABN and PNAT patients had similar enzymatic activity, including the stage of initiation of polymerization. In conclusion, enzymatic activity of RdRp was not responsible for the hepatocyte death that generates high levels of liver enzymes. At the same time, specific activity of RdRp was found to correlate with viremia and fibrosis. These data demonstrated that chronic hepatitis C patients with persistently normal liver enzyme levels should be followed-up and treated as patients with elevated liver enzyme levels because they can harbor actively replicating virus causing significant liver disease.

The second section focused on the **Immunometabolism of chronic viral infections and cancer.** The section was opened by the Plenary Lecture of Ujjwal Neogi (Department of Laboratory Medicine, Karolinska Institute, Stockholm, Sweden) on *Immune-Metabolic Characterizations in HIV-Infection – from Elite Controllers to Patients on Long-Term Successful Therapy.* HIV-1 elite controllers (EC) are a rare heterogeneous group of HIV-1-infected individuals who can suppress viral replication in the absence of antiretroviral therapy (ART). The mechanisms of how EC achieve undetectable viral loads are still unclear. Host plasma and faecal metabolomics and targeted plasma proteomics in a Swedish HIV-1 cohort including EC and treatment-naive viremic progressors (VP) as well as HIV-negative individuals (HC) have been investigated to get insights into EC phenotype. Plasma metabolites belonging to antioxidant defense had higher levels in EC relative to VP, whereas inflammation markers were increased in VP compared with EC. Only four plasma proteins (CCL4, CCL7, CCL20, and NOS3) were increased in EC compared with HC. CCL20/CCR6 axis can play an essential role in EC status [20]. Fecal metabolome and microbiome analysis showed an enrichment of dipeptides in EC compared to the other two study groups. The in silico and in vitro analyses identified anti-HIV-1 properties for two dipeptides that could bind to the HIV-1 gp120 and act as an HIV-1 antagonist. Furthermore, these dipeptides supported in vitro growth of the bacterial genus *Prevotella* enriched in EC, pinpointing its influence on the host metabolism. Thus, increased levels of both dipeptides and *Prevotella* could provide beneficial effects for EC [21]. Given that EC is a rare group of individuals, people living with HIV (PLWH) with long-term treatment were also analyzed. Alterations in the plasma metabolic profiles were investigated by comparing PLWH on long-term cART (>5years) and matched HIV-negative controls (HC) in two cohorts from low- and middle-income countries (LMIC), Cameroon, and India, respectively, to understand the system-level deregulation in HIV-infection. Using untargeted and targeted LC-MS/MS-based metabolic profiling and applying advanced system biology methods, an altered amino acid metabolism, in particular the one related to glutaminolysis, in PLWH compared to HC were reported. Another study combining both metabolomics and immune-phenotyping indicated altered glutamate metabolism associated with metabolic syndrome (MetS) in PLWH, which has clinical significance [22]. Further, modulation of cellular glutaminolysis promoted increased cell death and latency reversal in pre-monocytic HIV-1 latent cell model U1, which may be essential for the clearance of the inducible reservoir in HIV-integrated cells.

The second Plenary lecture of the section was delivered by David Finlay (Trinity College Dublin, Ireland). Prof Finlay spoke on *Metabolic Dysfunction of Natural Killer Cells in Cancer; Nutrients and Metabolites Play Their Part*. Natural Killer (NK) cells are key immune cells that can identify and kill tumor cells. However, they are often found to be dysfunctional in cancer patients. The metabolic requirements of NK cell and how these relate to the anti-tumor effector functions of these cytotoxic lymphocytes has been explored. This work has led us to an appreciation that both an excess or deficit of nutrients within a tumor microenvironment (TME) might cause NK cell dysfunction. When considering how the metabolic conditions within the TME can impact upon NK cells it is important to examine the nutrient transporters expressed on NK cells, such as Slc7a5 and CD71, and the activity of various nutrient sensing signaling molecules including mTORC1, cMyc and Srebp. The data supports the view of NK cell metabolism within the TME as a potential route towards improved of NK cell-based cancer immunotherapies.

The plenary was followed by the oral presentation by Maxim Nosenko (Trinity College Dublin, Ireland, previously at Engelhardt Institute of Molecular Biology, Moscow, Russia) who described how *Inflammation is Reprogramming Metabolic State of the Organism Via Accumulation of IL-6.* Inflammatory conditions are accompanied by profound adaptations of both cellular and systemic metabolism. Whereas immune cell metabolism is extensively investigated, little is known about the mechanisms of physiological metabolic shifts in the context of inflammation. Nosenko M et al employed mouse model of LPS-induced systemic inflammation that results in a drastic reprogramming of metabolic state of the organism and demonstrated the key role of IL-6 in regulation of this adaptation. Genetic or pharmacological inactivation of IL-6 significantly abrogated expression of key genes active in glycogen metabolism pathway regulating inflammation in the liver. This resulted in significant hypoglycemia and hypothermia in response to inflammatory conditions, that were not observed if IL-6 was inactivated. The involvement of glycogen metabolism pathway in regulation of glucose homeostasis during inflammation was further confirmed by pharmacological inhibition of glycogen phosphorylase. Finally, Nosenko M et al. found that IL-6 knock-out mice failed to induce fatigue upon LPS challenge as evidenced by RotaRod training. Fatigue, along with other well-described symptoms of so called “sickness behavior”, could potentially contribute to the energy balance shift towards immune response rather than physical activity. Authors believe that via regulation of systemic metabolism IL-6 launches crucial physiological adaptations to the inflammatory conditions. Altogether their data demonstrate specific role of IL-6 in regulation of systemic metabolism and physiology in the context of inflammation. Further unraveling of the mechanisms of these adaptations may potentially lead to the development of new therapies for infection- and cancer-induced multi-organ pathologies such as cachexia.

Anisov Denis (Englelhardt Institute of Molecular Biology, Moscow, Russia) presented data on *Itaconate and Dimethyl Itaconate Promote LPS-Induced Expression of IL-6 in White Adipose Tissue.* Itaconate (ITA) is an anti-inflammatory metabolite, a product of the decarboxylation of the TCA cycle intermediate cis-aconitate. ITA has recently attracted the attention due to its broad immunomodulatory properties linked to immunological tolerance. The itaconate pathway is a central regulatory node that links innate immune tolerance and trained immunity [23]. Anisov D et al have shown that treatment with dimethyl itaconate (D-ITA) restricts TNF and IL-6 production in LPS-activated murine bone marrow-derived macrophages (BMDMs). On the opposite, in vivo treatment with D-ITA and ITA increases the levels of IL-6 in the blood of LPS-treated mice. In addition, treatment of mice receiving LPS with ITA or D-ITA led to increased production of IL-6, IL-10 and CXCL2, decreased production of IFN-γ in the blood, as well as in the white adipose tissue. To conclude, the data by Anisenko et al. showed differential effects of ITA and D-ITA on inflammatory response in vitro and in vivo. Physiological functions of ITA and of ITA derivatives and their potential in therapeutic implications warrant further investigation

## Session 5: “Approaches to viral infection and cancer cure”

The last fifth session of the symposium reviewed existing approaches to cure chronic viral infections and associated cancer. The first section on **Novel viral infection and cancer treatments** was opened by the Plenary Lecture of Vladimir Chulanov (National Medical Research Center of Tuberculosis and Infectious Diseases, Moscow, Russia) on the advances in *Virological Cure of Chronic Hepatitis B Infection, from “Is It Possible?” to “When?”*. Chronic hepatitis B virus infection affects millions of people worldwide. Current treatment options can help to control viral infection and substantially reduce the risks of cirrhosis and hepatocellular carcinoma, but cannot eliminate the virus from infected cells and rarely achieve the so-called functional cure. A plethora of highly effective antiviral regimes and approaches are currently under development, including those targeting HBV transcription (i.e. siRNAs, SMC5/6, HBx, selective inhibitors of histone acetyltransferases), cccDNA (site-specific nuclease, cccDNA destabilizers), HBsAg release, HBcAg proteins, immune modulation (i.e. agonists of Toll-like receptors, anti-PD1 antibodies, cytokine therapies, CAR T cells), and approaches based on novel therapeutic vaccines. The lecture focused on different therapeutic strategies and the possible drawbacks. HBV cccDNA degradation and mutational inactivation can be efficiently destroyed by site-specific nucleases. It leaves out the issues of cleavage of integrated HBV, and of possible off-target mutagenesis. Transcriptional inactivation of HBV cccDNA may result in durable control of viral replication. The question remains if this effect is sustained, and if it is possible to achieve such control in humans. Prof. Chulanov concluded that the openings for HBV control and functional cure of infection in CHB patients lie in combination of modern and new forthcoming antivirals alongside with the therapeutic vaccines based on the novel platforms.

In the following oral, Kostyusheva Anastasia (National Medical Research Center of Tuberculosis and Infectious Diseases, Moscow, Russia) presented her data on *Rebound of HBV Replication Followed by Transient Crispr/Cas9 Rnps and its Prevention by rcDNA Depletion.* The in vitro study developed and tested the efficiency of CRISPR/Cas9-based strategies to clear the virus from HBV-infected cells. Although CRISPR/Cas9 RNPs cleared over 99% of HBV intermediates by the 3^rd^ day post intracellular delivery, HBV rebounded recovering replication by the 14^th^ day. Treating HepG2 cells 1 day before CRISPR/Cas9 RNPs delivery and for 5 days post-delivery by HBV reverse transcriptase inhibitor lamivudine prevented rebound of HBV replication. Kostyusheva A et al concluded that destroying >99% of HBV recombinant covalently closed circular DNA (rcccDNA) was not sufficient for clearing HBV from the infected cells. HBV rcDNA may form cccDNA de novo leading to viral rebound. However, the replicating virus could be eliminated by depletion of HBV rcDNA or by blocking rcDNA→cccDNA conversion step.

Brezgin Sergey (National Medical Research Center of Tuberculosis and Infectious Diseases, Moscow, and Sirius University of Science and Technology, Sochi, Russia) presented the data on *Antiviral Activity of Apobec3a and Apobec3b Induced by dSaCas9-p300 Ribonucleoprotein Complexes.* Apolipoprotein B mRNA editing catalytic polypeptide-like (APOBEC) family of proteins play important roles in the innate immune response to viral infections by editing viral genomes. Proteins of this family APOBEC3A (A3A) and APOBEC3B (A3B) restrict replication of a broad range of viruses [24]. Aim of the study was to develop a method for transient activation of A3A and A3B factors and test their anti-viral activity in in vitro model of HBV infection. Brezgin S et al have shown that treatment with ribonucleoprotein complexes of nucleolytically-dead high-fidelity variant of Staphylococcus aureus Cas9 protein fused to p300 acetyltransferase core subunit (dSaCas9-p300 RNPs) resulted in a transient but robust activation of A3A and A3B genes. Intracellular delivery of dSaCas9-p300 RNPs effectively suppressed HBV RNA and DNA levels, and resulted in a substantial decline in HBsAg levels. This for the first time demonstrated the ability of dCas9 RNPs to potently activate target gene expression that in case of A3A and A3B genes led to significant decline in the level of HBV replication intermediates. Importantly, this indicates the capacity of dCas9 RNPs to effectively function after transient, single delivery and that intracellular delivery of Cas9 RNPs may be more effective than transient transfection or long-term lentiviral transduction.

Isabelle Chemin (Cancer Research Center of Lyon, INSERM, CNRS, Lyon, France) presented *Update on Hepatocellular Carcinoma (HHC) Breakthroughs, do carcinomas have an Achilles Heal.* Poly (ADP-ribose) polymerase (PARP) is a family of proteins involved in a number of cellular processes such as DNA repair, genomic stability, and programmed cell death. PARP inhibitors (PARPi) combined with radiotherapy are considered to be promising for treatment of cancer. HBV X protein (HBx) is known to deregulate cellular DNA damage repair via SMC5/6 degradation [25]. Dr Chemin addressed the efficacy of PARPi in treatment of HCC by assessing its effects, alone and together with radiation, on DNA repair in cell lines expressing HBx as a model of HBV infection. Expression of HBx by cells, significantly lowered the survival of PARPi-treated cells. They also looked into PARP expression profiles and DNA damage levels in HCC tissues by assessing phosphorylation of Ser139 residue of histone variant H2AX, forming γH2AX detected by specific antibodies as foci. Formation of γH2AX is a marker of early cellular response to the induction of DNA double-strand breaks [26]. PARP1 and PARP2 transcript levels were significantly higher in tumor than all peri-tumor and control tissues except NASH-associated tumors. The HBV/HCV/alcohol-associated tumor tissues studied had reduced H2AX but higher gammaH2AX protein levels providing evidence of increased DNA damage during liver disease progression. Treatment with PARPi was cytotoxic; cytotoxicity was significantly enhanced when combined with X-rays (2Gy). Greater impact was seen after treatment with Talazoparib compared to Veliparib. Analysis of transcriptome and of DNA loss indicated that the effect was driven by SMC5/6 loss. These proof-of-concept experiments by Chemin I et al support application of PARPi alone or combined with radiotherapy for treatment of HBV-associated HCC and warrant further in-depth investigation.

Sofia Nefedorova (Engelhardt Institute of Molecular Biology, Moscow) focused her presentation on *Evaluation of Metabolic Inhibitors as Complements to Virotherapy of Solid Tumors.* Authors have shown synergetic cytopathic effect of 2-deoxy-D-glucose (DDG) pretreatment and viral infection on primary glioblastoma 5522 and 4434, model glioblastoma U251 and A172, as well as Hela cells. Cytopathic effect was more pronounced than in case of single DDG pretreatment. The highest effect was observed after treatment of the primary glioblastoma cells with 2 mM DDG simultaneously with virus infection: the drug increased the oncolytic activity of the virus by 10 fold. Interestingly, the effect was cell line-specific: the drug did not enhance cell death in three other cell lines tested. Authors plan to test if other metabolic inhibitors have similar effect. They also plan to evaluate metabolic changes in cells during viral infection using Extracellular flux analyzer Seahorse (Agilent), to show changes in the rate of glycolysis and identify other metabolic inhibitors, which could be used to enhance the oncolytic effect. The in vivo effect could be further assessed in murine models of immunodeficient mice with primary glioma xenografts.

Borgoyakova Maria (State Research Center of Virology and Biotechnology “Vector”, Koltzovo, Novosibirsky Region, Russia) presented data on *Immunogenicity of Combined DNA/Protein Anti-Sars-Cov-2 Vaccine.* Immunization with combined DNA/protein vaccine resulted in significant stimulation of antibody response against Spike (S) protein of SARS-CoV-2 and RBD domain. Protein-DNA complex immunogen was represented by VLPs that carried plasmid pVAX-S, encoding full-size S protein as a core, covered with polyglucin:spermidine with the recombinant RBD protein on its surface. Antibodies raised by VLP immunization had higher neutralizing activity against the live virus than antibodies raised by immunization with plasmid DNA or protein alone (at least 10-fold as high as in the control groups). As for T-cell response, the highest levels of IFN-γ producing cells including CD4+ and CD8+ T-lymphocytes were shown in the group receiving pVAX-S. The levels of IFN-γ producing T-cells in mice receiving VLPs were two times lower than in DNA-immunized mice; no IFN-γ response was detected in mice immunized with S protein. This study demonstrated that complexing the different vaccine modalities in VLP improves the humoral immune response to the vaccine, including the induction of virus neutralizing antibodies. Plasmid pVAX-S packed into the polyglucin-spermidine-RBD conjugate can be considered as a promising platform for developing new generation of anti-SARS-CoV-2 vaccines.

The last section of the symposium was devoted to **(Re)profiling of immune response with adjuvants.** It was opened by Alexandre B. Corthay (Dept. of Pathology, Oslo University Hospital, and SFF-Hybrid Technology Hub, University of Oslo, Norway) who gave a Plenary Lecture on *The Type of Immune Response is a Critical Factor in the Immune Battle Against Cancer.* The immune system has developed three main types of immune responses (type 1, 2, and 3) to optimally respond to different categories of pathogens and cancer. Each type of immune response is driven by a dedicated T helper (Th) cell subset. Th1 cells govern type 1 immunity that is protective against intracellular microbes and is also considered to be the most appropriate response to fight cancer. Th2 cells orchestrate type 2 immunity against parasites and venoms. Th17 cells control type 3 immunity to eliminate extracellular bacteria and fungi. T follicular helper (Tfh) cells participate in both type 1 and type 2 immune responses, while T regulatory (Treg) cells prevent immunopathology by suppressing detrimental immune responses of any type. The Th composition of human solid tumors remains poorly characterized. Therefore, a four-color multiplex chromogenic immunohistochemical assay for detection of Th1, Th2, Th17, Tfh and Treg cells in human tumor sections has been established. Using this new method, Corthay’s group found that human primary lung tumors are Th2-skewed and contain numerous Treg cells. In his lecture, Corthay A presented the result of the study recently published by his group in Frontiers in Immunology [27]. The results on Th2-skewing T-helper cells in lung cancer suggest that the response rate of immunotherapy for cancer could be increased by reprogramming the type of immune response from a detrimental Th2 to a beneficial Th1 type.

Anna Zajakina (Latvian Biomedical Research and Study Centre, Riga, Latvia) gave a presentation on the application of *Recombinant Viral Vectors as Adjuvants and Modulators of Immune Response for Cancer Treatment.* Viruses are natural mobile genetic elements that have effective properties of transferring genetic information into living organisms. Due to their ability to induce potent multilateral immune responses, viral vectors have recently received renewed attention as a platform of choice for next generation vaccines and immunotherapy adjuvants. In the field of cancer immunology, the discovery of tumor-specific neoantigens to which there is no self-tolerance, as well as the approval of effective immune checkpoint inhibitors has led to the clinical development of novel vector-based cancer therapies. The use of cytokines as vaccine adjuvants has a unique value in obtaining the appropriate immune response and in ensuring a protective outcome, especially in low/non-responding and immunosuppressed individuals. Previous studies indicated that cytokines can predetermine antibody isotype, to enhance systemic T-cell immune response, as well as to induce stronger immunological memory. The vector-based delivery of immune-modulating cytokines potentially can help to overcome the limitations of cytokine direct administration, in particular their toxicity and their short half-life following in vivo administration. Recombinant vaccines based on alphaviruses have demonstrated both prophylactic and therapeutic efficacy in pre-clinical studies. The biological features of alphaviruses have made them ideal tool for vaccine adjuvant development. A broad host range, direct targeting of dendritic cells, lack of genome integration, cytoplasmic RNA amplification and high-level antigen expression are important advantages for the application of alphaviruses as genetic complementary vaccines. They have proven to be safe in Phase I clinical trials in melanoma and kidney carcinoma patients. Recently, the group of Anna Zajakina has designed a set of Semliki Forest virus (SFV) and Sindbis virus (SIN) vectors producing proinflammatory cytokines and peptides (IL-12, IFN-γ, TNF-α and anti-TGF-β peptides) and confirmed their antitumor potential in a mouse model of breast cancer [28]. Her group focused on the remodeling of immunosuppressive tumor environment to avoid chemoresistance and immunotherapy escape. They have shown that alphavirus-derived IFN-γ synergizes with a TLR2/1 agonist to induce nitric oxide production in macrophages and polarization of macrophages to M1 phenotype pointing at their (re)programming towards tumor inhibition. Importantly, application of an SFV vector itself inhibited tumor growth and led to intratumoral increase of CD8 T cells and a decrease of myeloid cell populations. In conclusion, the SFV vectors expressing cytokines as IFN-γ vector can be effectively used to raise a therapeutic antitumor T-cell response and inhibit infiltration of myeloid cell into treated tumors, increasing the effectiveness of treatment.

Korotkaja Ksenia (Latvian Biomedical Research and Study Center, Riga, Latvia) focused her short presentation on *Macrophage 3D Cultivation as a Promising Model to Study the Induction of Innate Immune Response Against Viral Infections and Cancer.* A 3D in vitro model has been developed based on ultra-low attachment plates. The model was applied for studying different macrophage phenotypes. The secretome, surface marker profile and secreted chemokine profile of 3D cultivated M0, M1 and M2 macrophages had been determined. The results presented by Korotkaja K et al show that macrophages can be successfully cultivated and polarized in the 3D model. Furthermore, the plasticity of macrophages has been investigated by establishing a re-polarization model. Authors revealed rapid alteration of the macrophage polarization state after the change of added polarization stimuli. At the same time, re-polarized macrophages were expressing markers of the new phenotype while continuing to express the markers of the previous one, i.e. a definite unambiguous polarization could not be reached, demonstrating macrophage plasticity and requirement of continuous presence of polarizing stimuli. The 3D in vitro model offered by the authors is a promising method for investigation of immune cell response to different stimuli that can be applied to test the effects of new compounds as potential immunotherapeuticals for treatment of infectious diseases and cancer.

Merkuleva Iuliia (State Research Center of Virology and Biotechnology “Vector”, Koltzovo, Novosibirsky Region, Russia) presented data showing that *SARS-CoV-2 RBD Conjugated with Polyglucinum-Spermidine and dsRNA Elicits Strong Immune Response in Mice.* The comparative analysis of the sera of RBD-immunized mice for RBD-specific IgG and neutralizing antibodies showed that conjugation of RBD with either Polyglucinum-Spermidine (PGS) or PGS mixed with dsRNA (PGS+dsRNA), or Al(OH)_3_ enhances specific antibody response. There was no significant difference between the groups immunized with RBD-PGS+dsRNA and RBD+Al(OH)3. However, RBD-specific IgG titers were 27-fold, and neutralizing antibody titers were 3-fold higher in the group immunized with RBD-PGS+dsRNA than the group immunized with RBD+PGS without dsRNA. Next, ICS assay for antigen-specific CD4+ and CD8+ T-cell secretion of IFN-γ in murine splenocytes showed specific cellular immune response in all RBD-PGS+dsRNA immunized animals. Thus, the comparative immunogenicity assessment of RBD conjugated with PGS or PGS+dsRNA, and RBD in combination with Al(OH)3 revealed that used as an adjuvant PGS+dsRNA significantly enhances specific humoral and cellular immune response to RBD protein in mice. Authors suggested to use conjugation of immunogen with PGS+dsRNA as an alternative to current adjuvants for RBD-based subunit vaccine formulations.

Symposium was concluded by the **Closing Lecture** of Prof Joon Rhee (Combinatorial Tumor Immunotherapy, Medical Research Center; Department of Microbiology, Chonnam National University Medical School, Chonnam, South Korea) who spoke on *How to Make Cancer Vaccines More Effective? Importance of Adjuvant Strategy.* Cancer vaccines are designed to enhance tumor-specific cytotoxic T lymphocyte (CTL) responses resulting in tumor eradication or progression retardation. Despite a sound scientific basis for therapeutic cancer vaccines, extensive efforts have failed to bring success in clinical applications. Anti-tumor immune response to tumor immunotherapy relies on the immunogenicity of the tumor that varies between different types of cancer and between cancers of the same type in different individuals. Cancer antigens are derived from normal human cells, i.e. are autoantigens, and due to this, are less immunogenic than microbial antigens. To create successful cancer vaccines, novel ways to potentiate host immune responses against these non-immunogenic or low immunogenic proteins should be identified. Many extensively-tried tumor associated antigens used as vaccines were unsuccessful in inducing sufficient tumor suppression. Next-generation sequencing and improved bioinformatics tools have enabled better identification of tumor specific neoantigens, which are more desirable immunogens because they result from somatic mutations of normal genes and are more immunogenic than tumor associated antigens. However, even cancer specific neoantigens are still significantly less immunogenic than microbial antigens. Strong impact on the immunogenicity can be made by other vaccine components, namely, the delivery system and the adjuvant. Cancer vaccines require incorporation of potent adjuvants and break-through approaches to vaccine design and delivery. Specifically, he reviewed the peptide- versus protein-based therapeutic cancer vaccines. Peptide-based vaccines are comparatively easy to design, but they should be matched with the host’s MHC class I types. Protein-based vaccines need no matching, but to induce CTL response they need to be targeted to proteasomal pathway of processing, which requires their introduction into the cytosol of antigen expressing cells. For this, Prof Rhee suggested the use of therapeutic cancer vaccines (TCV) targeting dendritic cells (DCs) that deliver antigens into the cytosol of DCs. Prof Rhee stressed the importance of armoring cancer vaccines with novel potent adjuvants and gave an example of such adjuvant, flagellin, a protein-based PAMP capable of changing the immunosuppressive character of the tumor microenvironment. Mechanisms underlying the adjuvant effect of flagellin were presented from binding to extracellular Toll-Like Receptor 5 (TLR-5) to activation of the intracellular NLRC4 inflammasome pathway. Novel approach of TCV was proposed for built-in adjuvants by creating fusion proteins of flagellin with tumor associated antigens. Such novel approaches of incorporation of adjuvants into TCV hold promise for a wide range of cancer vaccine types.

Presentations of the early carrier researchers, totally 23 from 6 countries, were evaluated by the team of independent experts recruited among the members of the Program committee and experienced researchers who attended the meeting. Five winners were selected. The first prize was awarded to Flora Mikaeloff, Karolinska Institutet, Stockholm, Sweden, for the study “Trans cohorts metabolic reprogramming towards glutaminolysis in long-term successfully treated HIV-infection: potential role in accelerated aging and latency reversal” (https://techvac.org/announcement/). Early carrier researchers, winners of the contest, received awards and vouchers for publications in CANCERS and INFECTIOUS AGENTS AND CANCER.

Abstracts of the symposium will be published in the supplementary issue of INFECTIOUS AGENTS AND CANCER [29] and selected studies, in the special issue of CANCERS (MDPI) https://www.mdpi.com/journal/cancers/special_issues/Chronic_Viral_Infections_and_Cancer. Both journals acted as symposium partners and sponsors.

TECHVAC plans to continue research dissemination and education activities, aiming to organize virtual conference on vaccines and vaccination late autumn 2022. The announcement will appear at the Network site www.techvac.org.
